# Caveolin-1 is involved in encephalomyocarditis virus replication in BHK-21 cells

**DOI:** 10.1186/s12985-021-01521-3

**Published:** 2021-03-24

**Authors:** Qiongyi Li, Yang Liu, Shujuan Xu, Kexue Zhao, Ying Ling, Rongxiu Liu, Amjad Ali, Jialin Bai

**Affiliations:** 1 Biomedical Research Center, Key Laboratory of Biotechnology and Bioengineering of State Ethnic Affairs Commission, Northwest Minzu University, Lanzhou, China; 2 College of Life Science and Engineering, Northwest Minzu University, Lanzhou, China

**Keywords:** Endocytosis, Encephalomyocarditis virus, BHK-21, Caveolin-1, Clathrin, Macropinocytosis, Dynamin, Actin

## Abstract

**Background:**

Encephalomyocarditis virus, member of *Cardiovirus* genus within *Picornaviridae* family, is an important pathogen that infects different domestic and wild animals. However, the molecular mechanism of its entry remains unclear. In this study, we investigated the mechanism of EMCV infectivity in relation to endocytic pathway using BHK-21 cells.

**Methods:**

The function of numerous cellular key factors implicated in the various endocytic mechanisms were systematically explored using chemical inhibitors. Furthermore, RNA interference (RNAi) as well as the overexpression of dominant protein combined to virus infectivity assays, and confocal microscopy was used to examine EMCV infection in details.

**Results:**

The results indicated that the EMCV entry into BHK-21 cells depends on caveolin, dynamin, and actin but not clathrin nor macropinocytosis pathways. The effects of overexpression and knockdown of caveolin-1, one components of the caveolae, was examined on EMCV infection. The results showed that EMCV infection was positive correlation with caveolin-1 expression. Confocal microscopy analysis and internalization assay showed that caveolin-1 is required at the early stage of EMCV infection.

**Conclusions:**

Caveolin-1, dynamin, and actin-dependent endocytosis pathways are necessary for EMCV infection in vitro.

## Background

Encephalomyocarditis virus (EMCV) is a single-stranded, positive sense RNA virus of the *Cardiovirus* genus within *Picornaviridae* family that causes a wide range of diseases in mammalian species [1, 2]. After its first isolation from a captive male gibbon [3], the virus has been recovered from various animal species throughout the world [4–7]. EMCV is often used as a model to study antiviral immune responses, virus-induced myocarditis and insulin dependent diabetes mellitus [8, 9]. However, the mechanisms involved in the internalization and entry of the cardioviruses are still not well elucidated [2, 8].

Many viruses can trigger internalization by activating endocytic process such as macropinocytosis, clathrin-mediated endocytosis (CME) and caveolar/lipid raft-dependent, or clathrin- and caveolae-independent endocytic pathways are utilized by different viruses for their entry and replication in permissive cell [10–12]. Members of *Picornaviridae* family use different endocytic mechanisms for infection to occur. Pietia¨inen et al. in their study indicated that EV1 entry to CV-1 cells is dynamin-dependent [13]. Poliovirus infects different host cells using different endocytic pathways, for example, its entery into Hela cells is clathrin- and caveolin-independent while infects brain microvascular endothelial cells, it utilizes caveolin- and dynamin-dependent routes [14–16]. CBV3 enters into HeLa cells using clathrin-mediated endocytosis pathway [17] and coxsackie virus A9 entry into A549 cells is mediated by dynamin, β2-microglobulin, and Arf6 [12] while FMDV internalization is by clathrin-dependent pathway [18–20]. However, as one member of this family, little is known about the entry mechanism of EMCV.

Based on the above mentioned studies and being a member of the *Picornaviridae* family, we hypothesized that EMCV may also use the endocytic mechanisms for causing infection. Therefore, we designed this study to investigate EMCV infection with relation to endocytic pathway using BHK-21 cells. At first, we confirmed that the EMCV replication was related to endocytosis. Subsequently, we demonstrated that neither clathrin nor macropinocytosis pathway was involved in virus infection. We have shown that EMCV replication into the BHK-21 cells via caveolin-mediated and dynamin, actin-dependent pathway.

## Methods

### Cells, viruses, and antibodies

BHK-21 cells were provided by the Gansu Tech Innovation Center of Animal Cell (Northwest Minzu University, Lanzhou, China) and were maintained in DMEM (Minhai Bio-engineering, Lanzhou, China) supplemented with 10% FBS (Minhai Bio-engineering) in a 5% CO_2_ incubator at 37 °C.

The EMCV strain used in the current study was the BHK-21 cells adapted EMCV (GenBank: X74312) and its titer was 10^6.0^ TCID_50_ ml^−1^. The plaque-forming unit (PFU) was calculated as previously described [21].

Mouse monoclonal antibody (mAb) against Caveolin-1 and rabbit polyclonal antibody (pAb) anti-Caveolin-1 were bought from Beyotime (Beyotime Biotechnology, Shanghai, China) and MAb against glyceraldehyde-3-phosphate dehydrogenase (GAPDH) was purchased from Abcam (Abcam, Cambridge, UK). MAb against EMCV-VP1 was kindly donated by Dr. Juan Bai (College of Veterinary Medicine, Nanjing Agricultural University, China). Anti-GFP mAb were purchased from TransGen (TransGen Biotech, Beijing, China).The HRP-labeled secondary antibody, Alexa fluor-488-conjugated anti-mouse and Cy^TM^3-conjugated anti-rabbit IgG (H + L) were from Jackson ImmunoResearch Laboratories (Jackson ImmunoResearch Laboratories, PA, USA).

### qRT-PCR

Total RNA was isolated, followed by qRT-PCR as previously described [22], using primers EMCV-3D qF: GTCATACTATCGTCCAGGGACTCTAT and qR: CATCTGTACTCCACACTCTCGAATG. GAPDH was used as the internal reference and quantified using specific primers; qF: AAGGCCATCACCATCTTCCA and qR: GCCAGTAGACTCCACAACATAC.

### Gene overexpression and RNA interference

To evaluate the effect of Caveolin-1 in the infection as well as invasion of EMCV into BHK-21 cells, the replication-defective lentivirus system provided by Dr. Enqi Du (Northwest A&F University, China), was used to construct a recombinant plasmid to overexpress caveolin-1. Total RNA was extracted from BHK-21 cells and reverse transcribed into cDNA. The caveolin-1 gene was amplified by PCR based on the murine caveolin-1 sequence (GenBank accession No. U07645.1). The amplified PCR product was digested by restriction endonuclease XbaI and BamH I (NEB, MA, USA) and inserted into pTRIP-CMV-IRES-Puro to construct recombinant plasmid, pTRIP-CAV1, using specific primers (Table 1). As a control, a EGFP recombinant plasmid, pTRIP-EGFP, was also constructed. Lentivirus was produced with recombinant lentivirus vector pTRIP-CAV1 and pTRIP-EGFP as described [23] and the transfected cells were named as BHK-CAV1 and BHK-EGFP, respectively.

**Table 1:** The primer sequences used for Caveolin-1 overexpresses vector

Gene	Primer sequence (5′–3′)	Size (bp)
Caveolin-1	F:GCTCTAGAATGTCTGGGGGCAAATACGTGGACTC	537
	R:CGGGATCCTCATATCTCTTTCTGCGTGCTGATGC	

Underlined sequences show restriction enzyme sites (XbaI and BamH I) and start codon

Moreover, three individual small interfering RNAs (siRNA) against caveolin-1 (Rebobio, Guangdong, China) were designed (Table 2) and were employed to transfect the cells using Invitrogen Lipofectamine™ 2000 (ThermoFisher, MA, USA) system according to manufacturer's instructions. The silencing efficiencies were measured by qRT-PCR and Western Blotting (WB) analysis. After culturing for 2 days, the cells were infected with EMCV and at 9 h post-infection (hpi), WB and virus infectivity assays were performed.

**Table 2:** The sequence of the caveolin-1 siRNA

Name of siRNA	Sequence(5′–3′)
Mus-Cav1-siRNA1	GCAACATCTACAAGCCCAA
Mus-Cav1-siRNA2	CCACCTTCACTGTGACAAA
Mus-Cav1-siRNA3	CATCAAGAGCTTCCTGATT

### Chemical inhibitors and cell viability determination

DMEM supplemented with 10% FBS and one of the following chemical inhibitors: Nystatin, pitstop, dynasore, mitmab, cytochalasin D, jasplakinolide, chlorpromazine and 1, 1′-Dithiobis-2-naphthalenol (IPA-3) were purchased from Abcam. Methyl-β-cyclodextrin (MβCD) and NH_4_Cl were purchased from Sigma (Sigma, MO, USA) and bafilomycin A1, EIPA and wortmannin from Solarbio (Solarbio, Beijing, China). Concentration and effects of the inhibitors applied in this study were described in Table 3. Respective cells were treated with inhibitors for one hour before EMCV infection. After RNA interference and chemical inhibitors treatment, cells viability was conducted by the CellTiter 96® Aqueous Non-Radioactive Cell Proliferation Assay kit (Promega, WI, USA) according to the manufacturer’s instructions. The obtained raw values were converted to percentages in relation to untreated samples and corrected by background absorbance.

**Table 3:** Chemical inhibitors used in this study

Chemical inhibitor	Effect	Concentration
NH4Cl	Inhibits pH-dependent activation of the fusion protein	10 mM/20 mM
Bafilomycin A1	Vacuolar-type ATPase inhibitor	10 nM/20 nM
Chlorpromazine	Inhibits clathrin-dependent endocytosis	5 μM/10 μM
Pitstop-2	Cell-permeable clathrin inhibitor	5 μM/10 μM
Nystatin	Disrupts caveolae	12.5 μg/25 μg
MβCD	Extracts cholesterol from lipid membranes	2.5 mM/5 mM
Dynasore	Inhibits GTPase activity	20 μM/40 μM
Mitmab	Blocks the lipid binding	5 μM/10 μM
Cytochalasin D	Disrupts actin polymerization	5 μM/10 μM
Jasplakinolide	Stabilizes actin microfilaments	5 μM/10 μM
EIPA	NA^+^/H^+^ exchanger inhibitor	10 μM/20 μM
IPA-3	the Pak-1 inhibitor	7.5 μM/15 μM
Wortmannin	the PI3K inhibitor	5 μM/10 μM

### *Virus infectivity assays, post*-*entry inhibitory effects and detection of virus internalization*

For virus infectivity assays, cells were incubated with EMCV at 0.1 multiplicity of infection (MOI) for 1 h at 37 °C in serum-free medium and then washed three times with pre-warmed phosphate-buffered saline (PBS) and maintained in DMEM with 3% FBS. At the given time points post infection, the virus replication assay was examined by virus yield titration [24], qRT-PCR and western blotting.

For post-entry inhibitory effects determination, cells were first incubated with EMCV at 0.1 MOI. Then after two hoursrs, cells were washed with PBS and culture medium containing chemical inhibitors was added to the cells accordingly. At 9 h post infection, cells were harvested for further analysis [25].

EMCV internalizing ability into BHK-21, BHK-Cav1 and BHK-EGFP cells was determined by measuring the quantity of infectious viruses in these cells according to previous report [26].

### IFA and confocal microscopy

Cells were fixed using ice cold 75% ethanol at 4 °C for 30 min. For co-localization studies, cells were permeabilized with 0.1% Triton X-100 when needed. After washing cells on slides with PBS, the suitable primary antibody was added and incubated at 37 °C. 1 h later, the slides were washed again and 150 μl secondary antibody was added and incubated for 1 h. Finally, the samples were counterstained with 4, 6-diamidino-2-phenylindole (DAPI) for 5 min at room temperature and were analyzed under Confocal microscope ZEISS LSM 900 (Zeiss, Oberkochen, Germany).

### Western blotting

Samples were lysed in NP-40 lysis buffer (Beyotime, Shanghai, China) and concentration was measured using the Pierce BCA Protein Assay Kit (Thermo Fisher Scientific, A, USA). Samples were heated at 95 °C 5 min and run on 10% SDS-PAGE gel and were transferred to PVDF membrane (Millipore, MA, USA). After treatment with 5% milk for 1 h, the membrane was incubated overnight with the primary antibody at 4 °C and then treated with HRP-conjugated secondary antibody for 2 h at room temperature. The specific bands of the membrane were analyzed using chemiluminescence (Cowin Bioscience, Beijing, China) and detected using a Electrophoresis gel imaging split system (Gel imaging system, GE Healthcare Bio-Sciences AB). Protein ladders (10 kDa-180 kDa) used in this study was from YEASEN (Yeasen Biotech, Shanghai, China).

### Statistical analyses

Results are from three independent experiments and were analyzed with one-way ANOVA using Graphpad PRISM Version 5.0. Data was shown as the means ± standard deviations (SD). Differences were considered statically significant if *P*-value was less than 0.05 (*P < 0.05; **P < 0.01; ***P < 0.001).

## Results

### The role of endocytosis in EMCV replication in BHK-21 cells

**Fig. 1:**
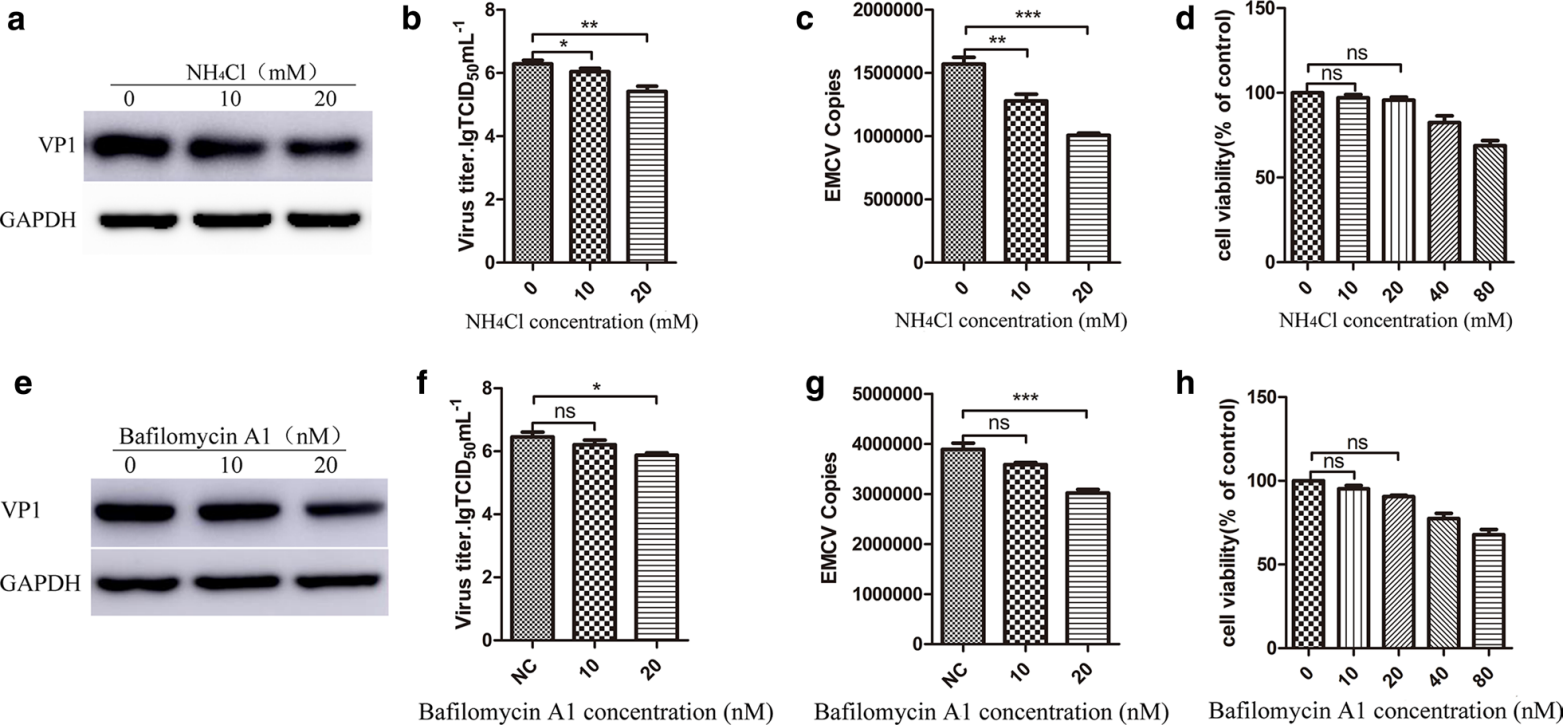
Inhibition of endosomal acidifcation with NH_4_Cl or Baflomycin A1 restrained EMCV replication in BHK-21 cells. NH_4_Cl treatment inhibited EMCV infection. BHK-21 cells were treated with NH_4_Cl, followed by incubation with EMCV at MOI of 0.1. At 9 h post infection (hpi), cells lysates or culture medium was collected, and virus infectivity assay was measured by WB (**a**), endpoint titration (**b**) and qRT-PCR (**c**). Cell viability assay was performed to evaluate the cytotoxicity of the NH_4_Cl (**d**). Baflomycin A1 (20 nM) treatment halted EMCV replication. BHK-21 cells were treated by Baflomycin A1 as indicated above and infectivity was measured by WB (**e**), endpoint titration (**f**) and qRT-PCR (**g**). Cell viability assay was performed to evaluate the cytotoxicity of Baflomycin A1 (**h**). Cultures treated with medium only were used as negative controls

To elucidate whether the endocytic pathway correlated with EMCV replication in BHK-21 cells, endocytosis specific inhibitors were used. The suitable non-toxic concentration of NH_4_Cl and Bafilomycin A1 were measured by the CellTiter 96® Aqueous Non-Radioactive Cell Proliferation Assay kit (Fig. 1d, h).

Previous research communicates that NH_4_Cl can hamper the endosomal entry of viruses by preventing pH-dependent activation of the fusion protein and by blocking membrane fusion [27]. In this experiment, BHK-21 cells were first treated with NH_4_Cl and then incubated with the virus. Afterwards; the media was changed to remove unbound viruses. EMCV-infected cells or culture fluids were harvested at 9 h post infection. As shown in Fig. 1a–c, we found that expression of VP1, EMCV-3D and virus titer were significantly decreased in infected cells compared to control cells in dose dependent manner. This indicated that EMCV infection is sensitive to inhibition of endosomal acidification.

Another inhibitor of endocytic compartment acidification, bafilomycin A1, was also tested [28]. Our results showed that 10 nM of bafilomycin A1 cannot block virus replication, while 20 nM of bafilomycin A1 can inhibit the proliferation of EMCV (Fig. 1e–g).

Next, we examined which endocytic pathway, including the clathrin-dependent pathway, macropinocytosis and caveolea-dependent pathway [12, 29], was utilized by EMCV to infect BHK-21 cells.

### Clathrin-mediated endocytosis is not involved in EMCV replication in BHK-21 cells

**Fig. 2:**
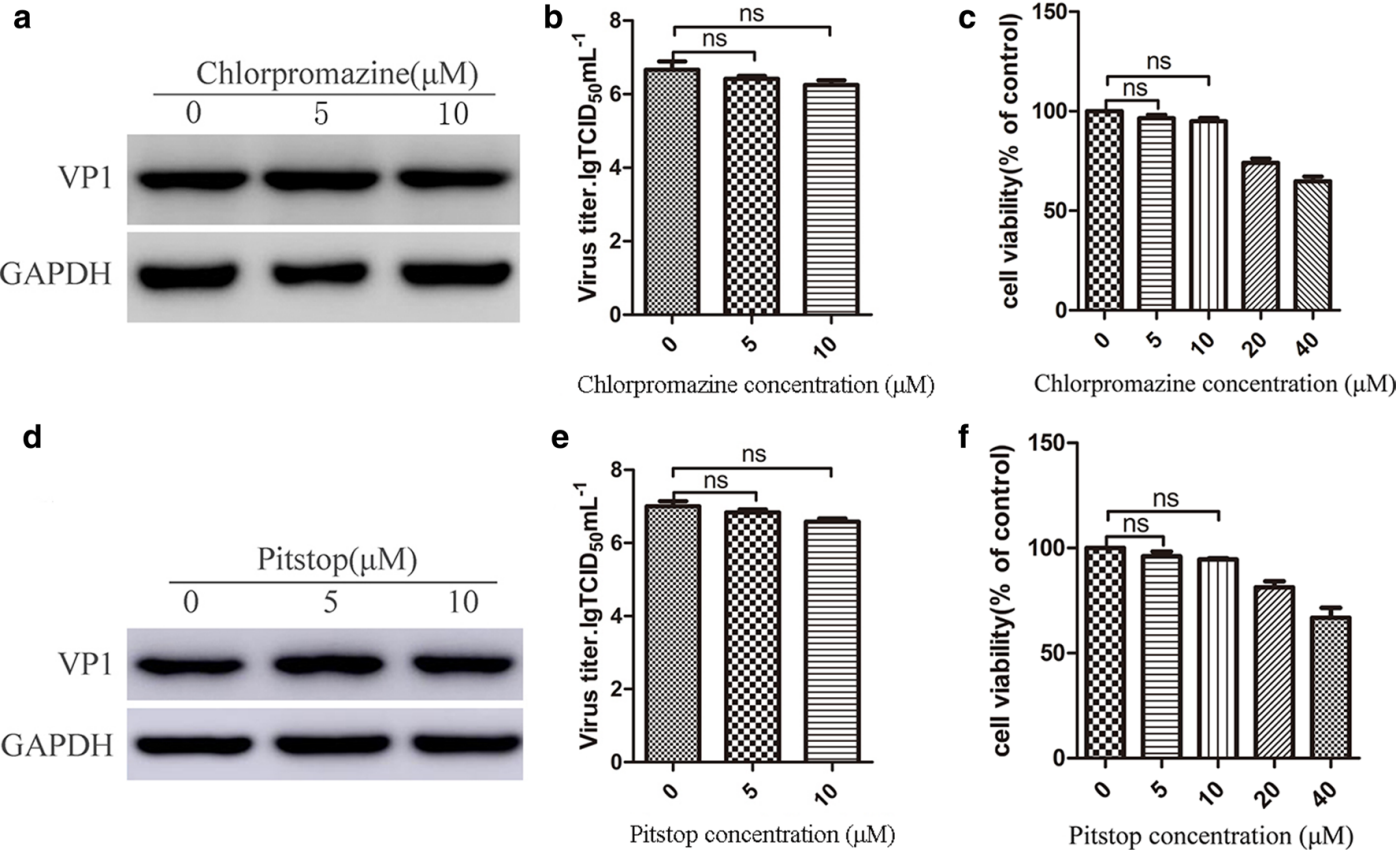
Clathrin-mediated endocytosis was not involved in EMCV infection Chlorpromazine treatment had no effect on EMCV replication. BHK-21 cells were treated with chlorpromazine, followed by incubation with EMCV at 0.1 MOI. At 9 hpi, cellular lysates or culture medium was collected, and virus replication assay was assessed by WB (**a**) and endpoint titration (**b**). Cell viability assay was performed to evaluate the cytotoxicity of the chlorpromazine (**c**). Pitstop-2 had also no effect on EMCV replication. BHK-21 cells were treated by Pitstop-2 as indicated above and infectivity was assessed by WB (**d**) and endpoint titration (**e**). Cell viability assay was performed to evaluate the cytotoxicity of the Pitstop-2 (**f**). Cultures treated with medium were used as negative control

As Clathrin-mediated endocytosis is often related to endosomal acidification [30] and is a classical pathway for most viruses to enter into host cells [11]. Therefore, we next detected whether EMCV enters into BHK-21 cells through clathrin-mediated endocytosis by using chlorpromazine and Pitstop-2 inhibitors [31, 32]. The desirable non-toxic concentration of inhibitors for cells was achieved as indicated (Fig. 2c, f). Analysis indicated that neither the expression of EMCV-VP1 (Fig. 2a, b) nor virus titer assays (Fig. 2d, e) were affected by chlorpromazine or Pitstop-2.

### EMCV replication in BHK-21 cells is independent of macropinocytosis

**Fig. 3:**
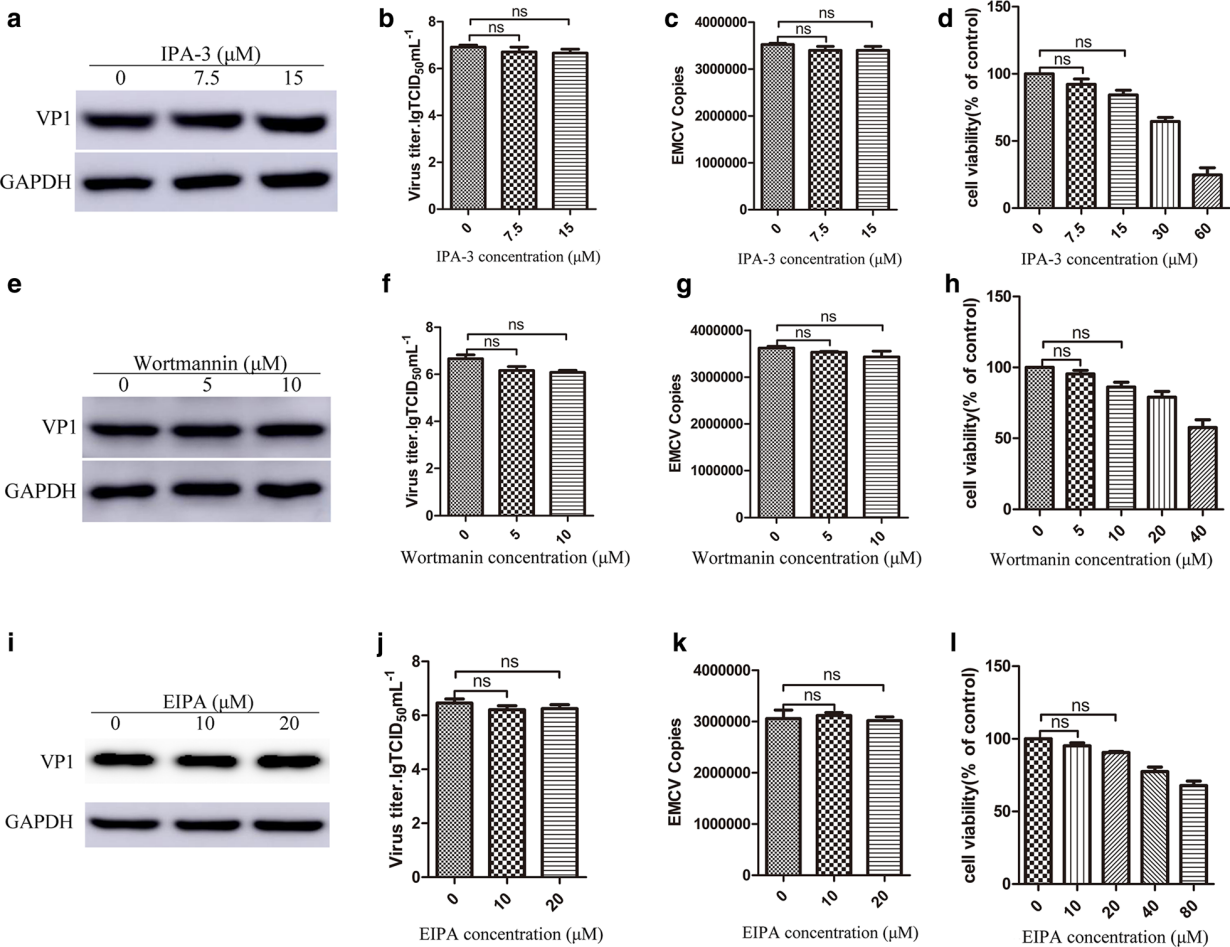
Macropinocytosis had no effect on EMCV replication. IPA-3 treatment did not inhibit EMCV replication. WB (**a**), endpoint titration (**b**) and qRT-PCR (**c**) analysis indicated that EMCV proliferation remained unchanged at 9 hpi when BHK-21 cells were treated with IPA-3. Cell viability assay was performed to evaluate the cytotoxicity of the IPA-3 (**d**). Wortmanin treatment did not inhibit EMCV replication as revealed by WB (**e**), endpoint titration (**f**) and qRT-PCR (**g**) at 9 h post treatment. Cell viability was measured before infectivity test (**h**). EIPA treatment had also no effect on EMCV replication as evident by WB (**i**), endpoint titration (**j**) and qRT-PCR (**k**). Cell viability was measured before infectivity test (**l**). Cultures treated with medium were used as negative control

To check whether the virus replication is macropinocytosis-mediated, BHK-21 cells were treated with NA^+^/H^+^ exchanger inhibitor EIPA, Pak-1 inhibitor (1,1′-Dithiobis-2-naphthalenol, IPA-3), and PI3K inhibitor, wortmannin [33]. Virus infectivity assay showed that none of them affected virus replication (Fig. 3a, b, c, e, f, g, i, j, k).

### Caveolae is required for EMCV replication in BHK-21 cells

**Fig. 4:**
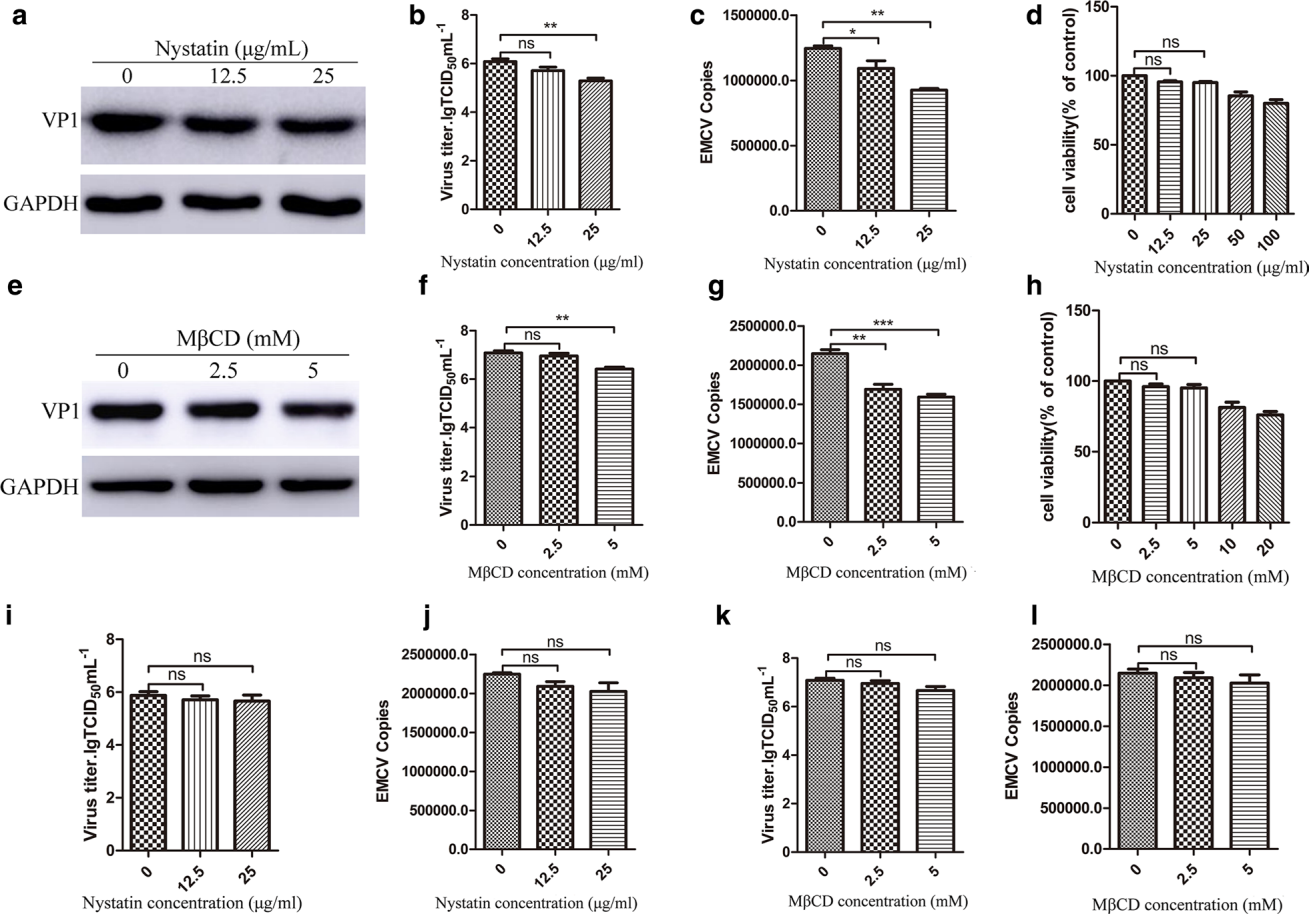
Caveolae is required for EMCV replication. Nystatin treatment inhibited EMCV replication. BHK-21 cells were treated with nystatin before the addition of the virus and virus infectivity assay was assessed at 9 hpi by WB (**a**), endpoint titration (**b**) and qRT-PCR (**c**). Cell viability assay was performed before experiments (**d**). MβCD treatment inhibits EMCV infectivity. Similar procedure like that of nystatin was adapted for MβCD. The analysis revealed that MβCD treatment inhibits EMCV infectivity as evident by WB (**e**), endpoint titration (**f**) and qRT-PCR (**g**). Cell viability assay was performed to evaluate the cytotoxicity of MβCD (**h**). Endpoint titration (**i**) and qRT-PCR (**j**) showed EMCV replication was no changed at 9 hpi when BHK-21 cells were treated with nystatin after virus was added. Similar procedure like that of nystatin was adapted for MβCD. The analysis revealed that MβCD has no effect on EMCV infectivity by endpoint titration (**k**) and qRT-PCR (**l**). Cultures treated with medium were used as negative control

Next, we investigated whether the caveolae-dependent pathway was involved in EMCV infection. Caveolae is rich in cholesterol and sphingolipids and can be disrupted by nystatin or MβCD [29]. The suitable non-toxic concentration was established (Fig. 4d, h). Results indicated that non-infected cell cultures when treated with certain concentration of nystatin (12.5 μg/ml, 25 μg/ml) and MβCD (2.5 mM, 5 mM) significantly inhibited EMCV proliferation (Fig. 4a, b, c, e, f and g**).** However, their effect on already infected EMCV-cell cultures was not significant (Fig. 4i–l).

### Caveolin-1 facilitates EMCV infection

**Fig. 5:**
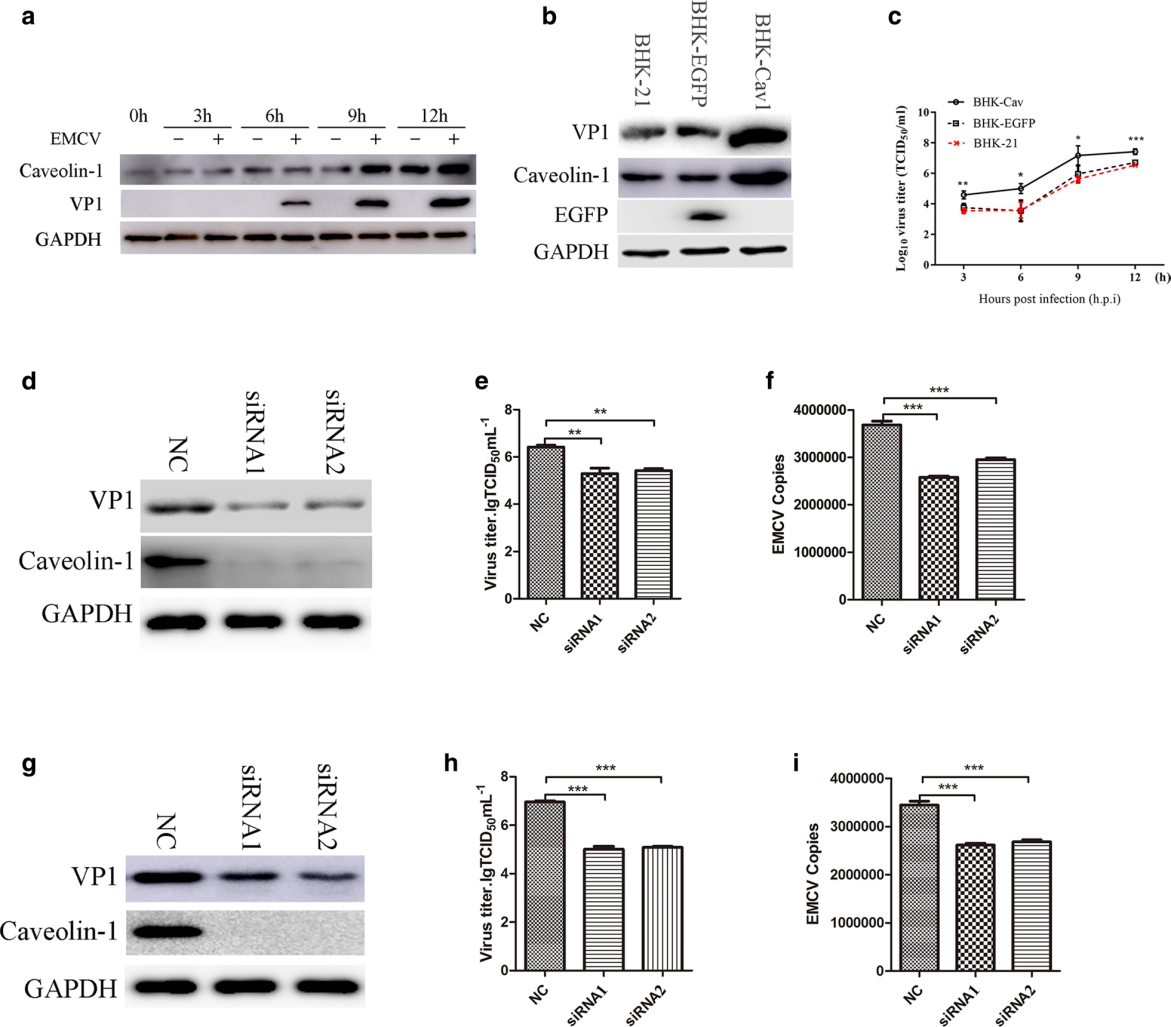
Caveolin-1 is associated with EMCV replication. Caveolin-1 was up-regulated with EMCV infection in time dependent manner (**a**). Increased viral protein production in BHK-Cav1(**b**). BHK-Cav1, BHK-EGFP and BHK-21 cells were infected with EMCV at 0.1 MOI and at 9 hpi, cellular lysates were collected and immunoblotted by using EMCV-VP1 specific antibodies. The blot was also stained with anti-caveolin-1, anti-GAPDH antibodies and anti-GFP antibodies. Growth kinetics of EMCV in BHK-Cav1, BHK-EGFP and BHK-21 cells (**c**). BHK-Cav1, BHK-EGFP and BHK-21 cells were incubated with EMCV at 0.1 MOI for 1 h at 37℃. After adsorption, the inocula were removed and cells were supplied with fresh medium. Cultures were harvested at 3-h intervals and virus titers were determined. The expression of EMCV-VP1 (**d**), virus titers (**e**) and copy number (**f**) were significantly reduced at 9 hpi in specific siRNA-transfected BHK-21 cells. Similarly, BHK-Cav1 cells were transfected with indicated siRNAs with reference to controls (NC). The expression of EMCV-VP1 (**g**), virus titers (**h**) and copy number (**i**) decreased significantly at 9 hpi in siRNA-transfected BHK-Cav1 cells

Caveolin-1 is the main structural protein of caveolae and is associated with the internalization of many viruses into their respective hosts [35]. In order to explore whether EMCV exploits caveolin-1 during its infection, the expression of caveolin-1 during EMCV infection was investigated. WB analysis indicated that caveolin-1 expression was increased in infected cells in a time-dependent manner, consistent with the expression of the EMCV VP1 protein (Fig. 5a).

To further investigate the impact of caveolin-1 on EMCV infection, overexpression of caveolin-1 was carried out in relation to EGFP and BHK-21 cells (Fig. 5b). BHK-Cav1 and BHK-EGFP cells were cultured in medium without puromycin at least for 2 weeks prior to EMCV infection. Then BHK-Cav1, BHK-EGFP and BHK-21 cells were incubated with 0.1 MOI EMCV at 37 °C for 1 h and cells were harvested for WB analyses at 12 h post infection.

As shown in Fig. 5b, the expression of VP1 was significantly higher in BHK-Cav1 cells compared to control cells. In order to confirm that EMCV replication is truly upregulated by the overexpression of caveolin-1, culture supernatants were collected at 3-h interval, and viral titers were determined as described previously [24]. The growth kinetics experiment showed that the overall process of virus replication was more efficient in BHK-Cav1 than in BHK-21 and BHK-EGFP cells (Fig. 5c).

For a more in depth understanding of the molecular pathogenesis of EMCV infection in vitro, knockdown experiments using specific or control siRNA sequences were conducted. RNA interference silenced caveolin-1 expression in BHK-21 cells, in turn, impacted viral infection process as evident by the expression of VP1 (Fig. 5d), virus titers (Fig. 5e) and virus copies number (Fig. 5f).

To further elaborate caveolin-1involvement in the infection process, the same siRNA experiment was repeated in BHK-Cav1 cells. Results indicated that down-regulation of caveolin-1 significantly inhibited the virus replication in BHK-Cav1 cells (Fig. 5g–i).

### Caveolin-1 is essential for EMCV infection by involving in internalization

**Fig. 6:**
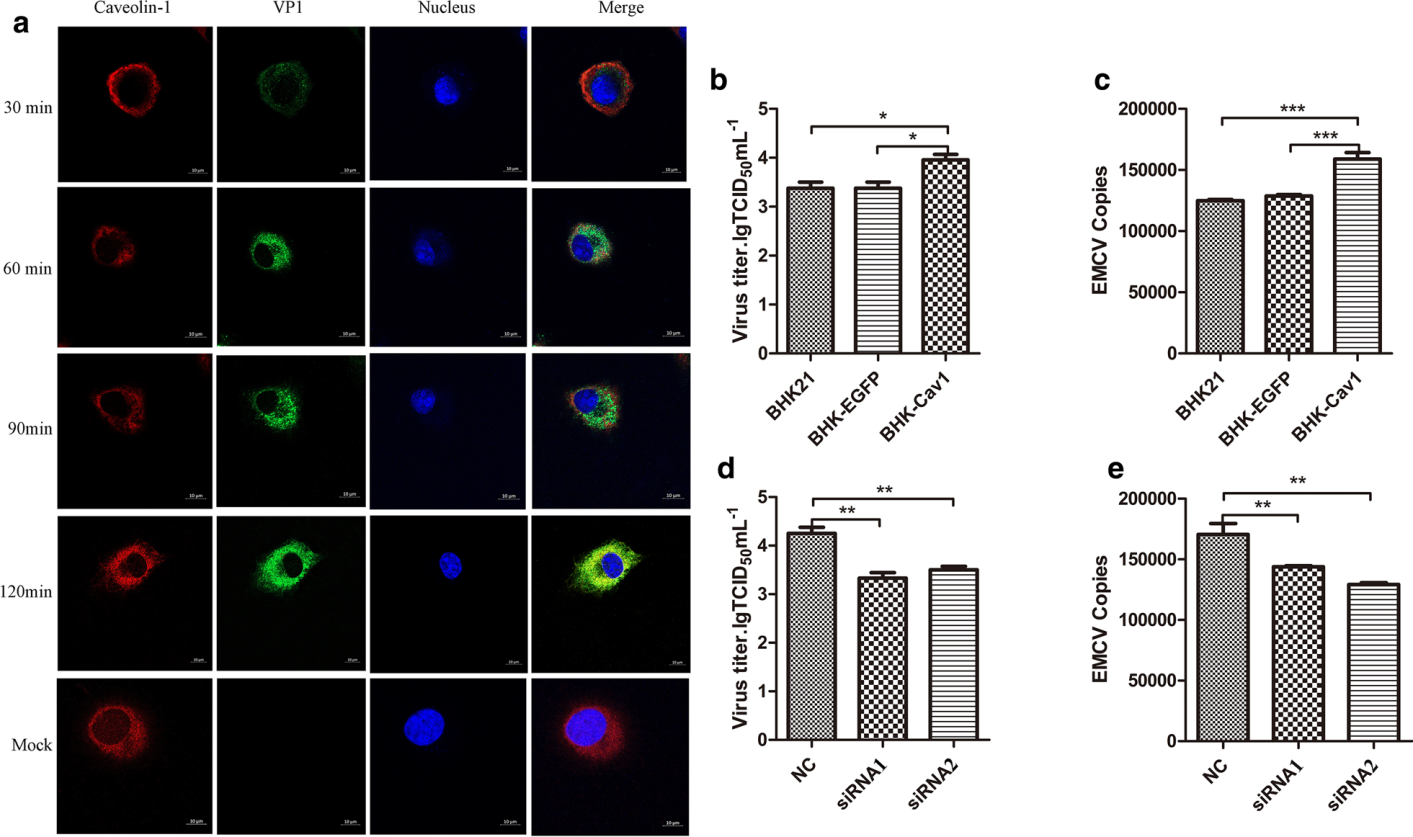
Caveolin-1 mediated EMCV internalization. EMCV-VP1 co-localized with caveolin-1 **(a)**. Cells treated with virus at MOI of 3 were synchronized on ice for 60 min and incubated at 37 °C for 0, 30, 60, 90 and 120 min before they were washed and fixed. Localization was analyzed by confocal microscopy (Scale bar = 10 µm) after performing a double immunofuorescence staining to visualize early caveolin-1 (left) and EMCV VP1 (middle). Right panel shows merged images; green indicates the virus and red indicates caveolin-1. Cell nuclei are indicated in blue while mock as a negative control. EMCV internalization was increased when caveolin-1 was overexpressed. BHK-Cav1, BHK-EGFP and BHK-21 cells were incubated with 3 MOI of EMCV at 37 °C for 1 h, harvested and cell lysates were prepared by three cycles of freeze-thawing. Virus titer assay (**b**) and qRT-PCR (**c**) analysis showed that EMCV internalization was increased in BHK-Cav1. EMCV internalization was decreased when caveolin-1 was down-regulated. BHK-21 cells transfected with negative-control (NC) siRNA and with two specific caveolin-1 siRNAs. Similar procedure was performed as mentioned above. Virus titer assay (**d**) and qRT-PCR (**e**) analysis revealed that virus internalization was decreased significantly in siRNA1 and siRNA2 group

To ensure the effect of caveolin-1, we examined the co-localization of virus with caveolin-1 by confocal imaging. As shown in Fig. 6a, after exposed to the EMCV for 120 min, EMCV-VP1 co-localized with caveolin-1 could be observed in infected BHK-21 cells.

As EMCV-VP1 co-localized with caveolin-1 at 120 min post infection, we next investigated caveolin-1 association with EMCV internalization. Increased EMCV internalization efficiency was noticed in BHK-Cav1 as compared to BHK-EGFP or BHK-21 cells (Fig. 6b, c). Consistent with the results of caveolin-1 overexpression, siRNAs that effectively restrained caveolin-1 expression and inhibited the EMCV internalization (*P* < *0.01*) as compared to control (Fig. 6d, e).

### Dynamin is needed for EMCV replication in BHK-21 cells

**Fig. 7:**
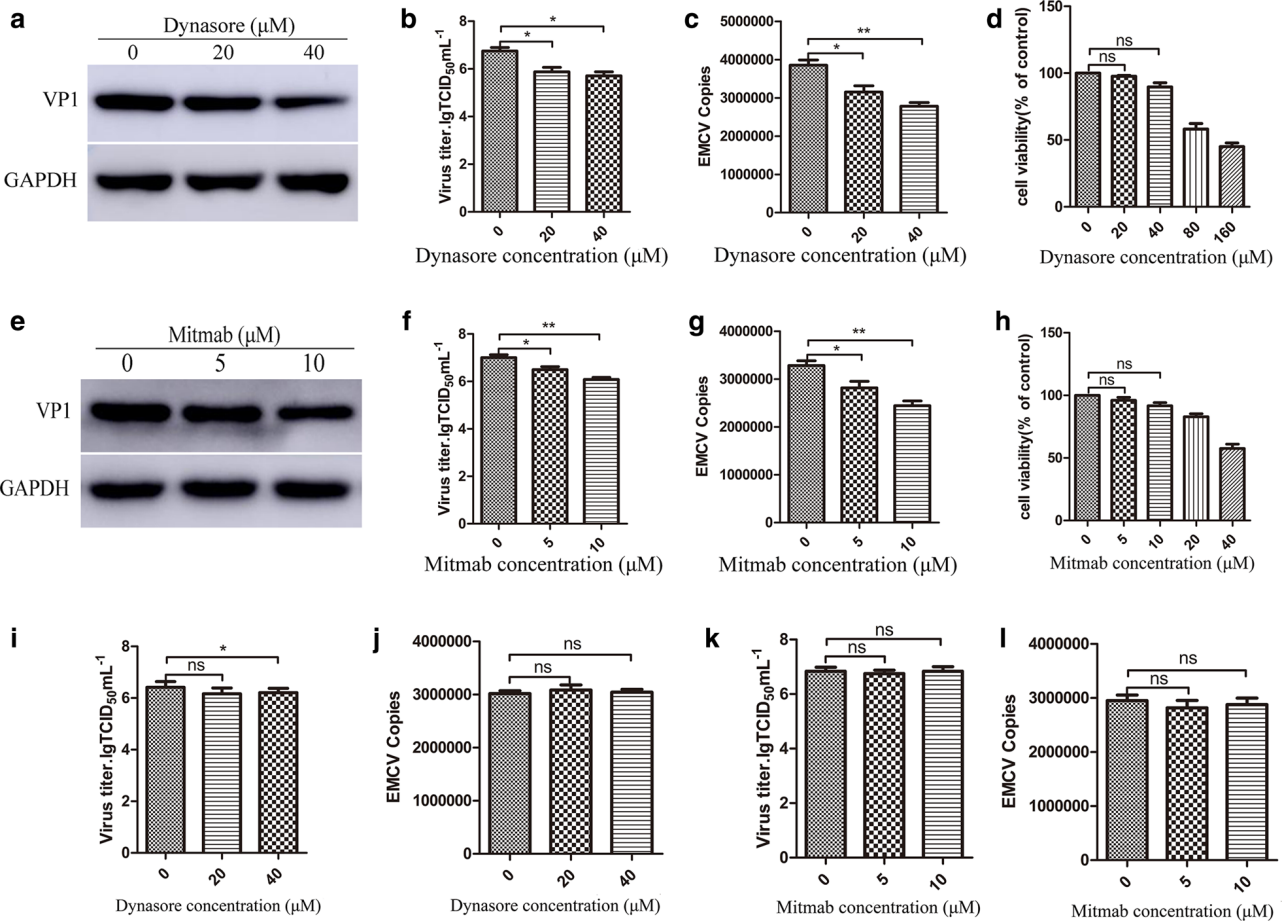
Dynamin inhibitors down regulated EMCV infection. Dynasore treatment inhibited EMCV replication. WB (**a**), endpoint titration (**b**) and qRT-PCR (**c**) showed EMCV replication was decreased at 9 hpi when BHK-21 cells were treated with dynasore before virus was added. Cell viability assay was performed before experiments (**d**). Mitmab treatment inhibited EMCV replication. WB (**e**), endpoint titration (**f**) and qRT-PCR (**g**) were performed at 9 hpi as indicated above. Cell viability was measured before infectivity test (**h**). Baflomycin A1 (20 nM) treatment halted EMCV replication. BHK-21 cells were treated by Baflomycin A1 as indicated above and infectivity was measured by WB (**e**), endpoint titration (**f**) and qRT-PCR (**g**). Post-BHK-21 infected cells treatments with dynasore had no effect on EMCV replication. Endpoint titration (**i**) and qRT-PCR (**j**) showed EMCV replication was no changed at 9 hpi when BHK-21 cells were treated with dynasore after virus was added. Similar procedure like that of dynasore was adapted for mitmab. The analysis revealed that mitmab has no effect on EMCV infectivity by endpoint titration (**k**) and qRT-PCR (**l**). Cultures treated with medium were used as negative control

Dynamin is a kind of large GTPase that can promote the split of endocytic membranes [12] and is considered to have a role in both clathrin-dependent endocytosis and several other endocytic pathways [34]. Therefore, we investigated its potential role in EMCV replication. Two inhibitors of GTPase activity, dynasore [35, 36] and the lipid-binding mitmab were selected [37], and their optimal concentrations were obtained by cell viability assay (Fig. 7d, h). Results indicated that these inhibitors significantly inhibited virus replication in BHK-21 before infection when introduced before infection (Fig. 7a, b, c, e, f and g) but their effect on infected cells was not significant when added after infection (Fig. 7i–l).

### Role of actin in EMCV infection in BHK-21 cells

**Fig. 8:**
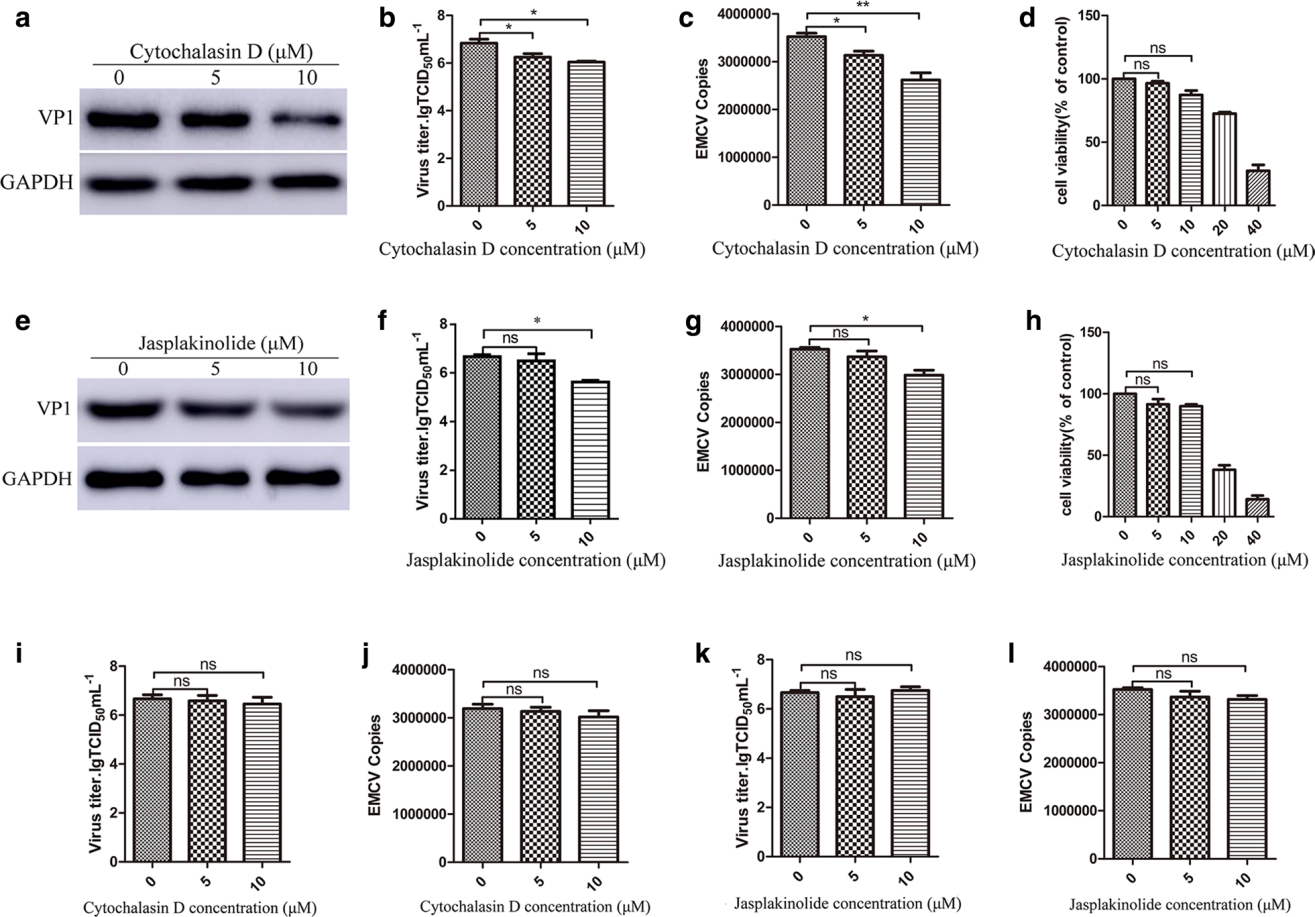
Cytoskeleton reorganization affects EMCV replication. Cytochalasin D treatment inhibited EMCV replication. WB (**a**), endpoint titration (**b**) and qRT-PCR (**c**) showed EMCV replication was decreased at 9 hpi when BHK-21 cells were treated with cytochalasin D before virus was added. Cell viability assay was performed before experiments (**d**). Jasplakinolide treatment inhibited EMCV replication. WB (**e**), endpoint titration (**f**) and qRT-PCR (**g**) at 9 hpi was performed as indicated. Cell viability was measured before infectivity test (**h**). Post-BHK-21 infected cells treatments with cytochalasin D had no effect on EMCV replication. Endpoint titration (**i**) and qRT-PCR (**j**) showed EMCV replication was no changed at 9 hpi when BHK-21 cells were treated with cytochalasin D after virus was added. Similar procedure like that of cytochalasin D was adapted for jasplakinolide. The analysis revealed that jasplakinolide has no effect on EMCV infectivity by endpoint titration (**k**) and qRT-PCR (**l**). Cultures treated with medium were used as negative control

Results of the current study suggested that EMCV infection in BHK-21 cells is mediated by caveolin- and dynamin-dependent endocytosis. Next, the role of the cytoskeleton during virus entry was examined by actin disrupting agent (cytochalasin D) and stabilizing compound (jasplakinolide) [38, 39]. The optimal concentrations of these two inhibitors were obtained by cell viability assay (Fig. 8d, h). We found that both actin-stabilizing jasplakinolide and actin-disrupting agent cytochalasin D significantly halted EMCV infection when introduced into cells before infection (Fig. 8a, b, c, e, f and g). However, the post-infected treatment was not significant (Fig. 8i–l).

## Discussion

Endocytosis is an important cellular process that mediates nutrient uptake, receptor internalization and the regulation of cell signaling (Endocytosis in proliferating, quiescent and terminally differentiated cells. 2018). For a large number of viruses, they can take advantage of the endocytosis machinery for infecting humans and animals (Endocytosis of Viruses and Bacteria, Pascale Cossart and Ari Helenius). Previous studies showed that members of *Picornaviridae* family use different endocytic mechanisms for entry into host cells [12, 15, 17, 19, 20]. However, the mechanisms involved in internalization of EMCV are poorly understand.

In the current study, we investigated the role of endocytosis in EMCV infection in BHK-21 cells. Lysosomotropic agents sensitivity is considered a good evidence of endocytosis [40]; therefore, we pretreated cells with different inhibitors (NH_4_Cl or Baflomycin A1) of endosome acidification. Both reagents partly inhibited the virus infectivity (Fig. 1), suggesting that endocytosis has a role in EMCV infection.

Earlier studies on both enveloped and nonenveloped viruses, such as HIV [41], adenovirus [42], foot-and-mouth disease virus [43], reovirus [44] and bluetongue virus [45, 46], document that the virus entry into their respective host cells is by clathrin-mediated pathway. But results of our study indicated that neither viral structural proteins nor virus titers were significantly decreased by treatment of cells with clathrin specific inhibitors (Fig. 2a, b, d, e) suggesting that clathrin-mediated endocytosis might not be an essential pathway for EMCV infection.

As one of the endocytic mechanisms in mammalian cells, macropinocytosis involves internalization of large number of plasma membrane together with extracellular medium and forms micropinosome [47]. Many intracellular pathogens by host cells via macropinocytosis have been described. Some viruses, such as African swine fever virus (ASFV) [48], Ebola virus [49] and Human cytomegalovirus (HCMV) [50], use this pathway to gain access to host cell [47]. Then we supposed whether macropinocytosis-dependent pathway may be involved in EMCV infection and hence first, cells were pretreated with macropinocytosis specific inhibitor such as EIPA, IPA-3, wortmannin and then infected with the EMCV. Our results showed that these inhibitors did not affect EMCV infectivity assay (Fig. 3a, b, c, e, f, g, i, j, k) and thus implies that macropinocytosis is not involved in EMCV infection in vitro.

Apart from clathrin-dependent endocytosis, lipid raft and caveolae-dependent endocytosis are alternative endocytic pathways proposed for viruses intake [51]. It has been reported that MβCD could inhibit the CAV9 infection via lipid microdomains [52], therefore, we examined the role of caveolar/lipid rafts endocytosis in EMCV infection in BHK-21 cells. We found that EMCV infection significantly decreased in MβCD and nystatin treated cells compared to control before incubated with EMCV (Fig. 4a, b, c, e, f, g), while their effect was limited when added after infection (Fig. 4i–l). These results indicated that caveolae is involved in early stage of EMCV replication.

Caveolin-1 is the main structural protein of caveolae and has various functions in endosomal membrane traffic and other cellular processes, such as endocytosis, signal transductions, protein trafficking and secretion [53–56]. Additionally, caveolin-1 is also involved in many viruses entry process, such as HIV [57], aquareoviruses [58], coronavirus [59], HCV [60], RSV [61] and CSFV [62]. The dependence of EMCV infection on caveolae-dependent pathway and the findings that EMCV infection corresponds to caveolin-1 expression level (Fig. 5a), directed us to study whether caveolin-1 is an important element involved in the replication process of the virus. To access the possibility, the lentiviral vector overexpressed caveolin-1 and siRNA targeted caveolin-1 were constructed. It is evident when overexpression of caveolin-1 resulted in a clear increase in the infection efficiency compared to the control cells (Fig. 5b, c). Conversely, decreased expression of caveolin-1 by siRNA, inhibited the virus replication in BHK-21 cells (Fig. 5d–i). The results highlight the importance of caveolin-1 for EMCV infection in BHK-21 cells.

Entry of viruses into permissive cells is an important stage in the viral pathogenesis [11, 51] and different viruses exploit various cellular endocytic mechanisms to initiate internalization and infection [63]. Results of the co-localization experiment in the current study at different time intervals suggested that there was co-localization of EMCV-VP1 and caveolin-1 at 120 min post infection (Fig. 6a) which implies that that caveolin-1 is required for early stage of EMCV replication. Further, EMCV internalization was enhanced by overexpression of caveolin-1 (Fig. 6b, c) while EMCV internalization was strongly inhibited when caveolin-1 was downregulated (Fig. 6d, e). These findings, together with the results of co-localization (Fig. 6a) indicated that caveolin-1 is required for the internalization and infection of EMCV in vitro.

Previous studies have pointed out that either overexpressed dominant-negative mutants of dynamin or disrupted actin assembly can block caveolae-mediated endocytosis [29, 64]. Therefore, we studied the potential role of dynamin and actin in EMCV infection in BHK-21 cells using two inhibitors of dynamin (dynasore and mitmab). Both inhibitors affected virus replication when added before infection (Fig. 7a, b, c, e, f and g). However, neither dynasore nor mitmab blocked EMCV infection when added after infection (Fig. 7i–l). Together, these results suggest that dynamin plays an exclusive role in virus uptake and thereby mediating infectivity.

It has been known that cytochalasin D inhibits actin subunits polymerization, whereas jasplakinolide inhibits the polymerization to stabilize the filaments [38, 65, 66] and we implemented this observation to determine their role in EMCV infection. We discovered that virus replication was decreased in cells pretreated with cytochalasin D and jasplakinolide (Fig. 8a, b, c, e, f and g) but their effect was limited when added after infection (Fig. 8i–l). This revealed that both actin filaments and actin reorganization are required for EMCV infection in vitro.

## Conclusion

In conclusion, the present study demonstrates for the first time that caveolin-1, dynamin and actin-dependent endocytosis pathways are involved in EMCV uptake, internalization and its subsequent replication in BHK-21 cells in vitro. Remarkably, there is a positive correlation between expression level of caveolin-1 and EMCV replication in vitro. Further work is needed to investigate the role of phosphorylation of caveolin-1, and the related singling pathway in regulating EMCV entry and replication.

## Data Availability

All data included in the manuscript are available.

## Abbreviations

EMCV: encephalomyocarditis virus
DMEM: Dulbecco’s modified eagle medium
FBS: fetal bovine serum
MEM: minimum essential medium
PFU: plaque-forming unit
WB: western blotting
mAb: monoclonal antibody
pAb: polyclonal antibody
qRT-PCR: quantitative reverse transcription polymerase chain reaction
IFA: indirect immunofluorescence assay
DAPI: 4,6-diamidino-2-phenylindole
MOI: multiplicity of infection
hpi: hour post infection
